# QTLs Controlling Physiological and Morphological Traits of Barley (*Hordeum* *vulgare* L.) Seedlings under Salinity, Drought, and Normal Conditions

**DOI:** 10.3390/biotech11030026

**Published:** 2022-07-15

**Authors:** Somayyeh Makhtoum, Hossein Sabouri, Abdollatif Gholizadeh, Leila Ahangar, Mahnaz Katouzi

**Affiliations:** 1 Department of Plant Production, Faculty of Agriculture Science and Natural Resources, Gonbad Kavous University, Gonbad 4971799151, Iran; somayyeh.makhtoum@yahoo.com (S.M.); lgholizadeh@gmail.com (A.G.); l.ahangar63@gmail.com (L.A.); 2 Crop Génome Dynamics Group, Agroscope Changins, 1260 Nyon, Switzerland

**Keywords:** barley, salinity, drought, molecular markers, marker assisted selection

## Abstract

To identify the genomic regions for the physiological and morphological traits of barley genotypes under normal salinity and drought, a set of 103 recombinant inbred line (RIL) populations, developed between Badia and Kavir crosses, was evaluated under phytotron conditions in a completely randomized design in 2019. Linkage maps were prepared using 152 SSR markers, 72 ISSR, 7 IRAP, 29 CAAT, 27 SCoT, and 15 iPBS alleles. The markers were assigned to seven barley chromosomes and covered 999.29 centimorgans (cM) of the barley genome. In addition, composite interval mapping showed 8, 9, and 26 quantitative trait loci (QTLs) under normal, drought, and salinity stress conditions, respectively. Our results indicate the importance of chromosomes 1, 4, 5, and 7 in salinity stress. These regions were involved in genes controlling stomata length (LR), leaf number (LN), leaf weight (LW), and genetic score (SCR). Three major stable pleiotropic QTLs (i.e., qSCS-1, qRLS-1, and qLNN-1) were associated with SCR, root length (RL), and root number (RN) in both treatments (i.e., normal and salinity), and two major stable pleiotropic QTLs (i.e., qSNN-3 and qLWS-3) associated with the stomata number (SN) and LW appeared to be promising for marker-assisted selection (MAS). Two major-effect QTLs (i.e., SCot8-B-CAAT5-D and HVM54-Bmag0571) on chromosomes 1 and 2 were characterized for their positive allele effect, which can be used to develop barley varieties concerning drought conditions. The new alleles (i.e., qLWS-4a, qSLS-4, qLNS-7b, qSCS-7, and qLNS-7a) identified in this study are useful in pyramiding elite alleles for molecular breeding and marker assisted selection for improving salinity tolerance in barley.

## 1. Introduction

After wheat, rice, and corn, barley (*Hordeum vulgare* L.) is a vital cereal worldwide. Its importance is related to its ability to grow in dry, low-temperature, and salinity conditions [1]. Barley is one of the major crops grown in drylands, with a low average yield. Due to the level of barley cultivation and global dryland expansion, it is necessary to search for the selection of drought-tolerant cultivars or genotypes to increase its yield [2].

Environmental stresses are among the main factors reducing the growth and yield of agricultural products worldwide. After drought, salinity is one of the most significant environmental stresses [3]. Barley is one of the most important species tolerant to environmental stresses. However, its growth and yield are strongly affected in many parts of the world, resulting in severe economic losses [4].

Identifying and selecting physiological or morphological traits related to plant performance under drought and salinity stresses is considered a valuable strategy [5]. Moisture and salinity stresses are lethal to barley seedlings [6]. The response of barley to drought and salinity stresses is very complex. The genetic study of tolerance to these stresses is also complex. Drought and salinity tolerance in barley seedlings is controlled by several genes [7,8,9]. Various seedling traits are significantly subjected to abiotic stress. Therefore, selection for drought and salinity tolerance based on plant phenotype is insufficient [10].

Important traits involved in drought and salinity tolerances are related to roots, stomatal structure, and chlorophyll. Among morphological traits, stomata are specialized epidermal structures that control the exchange of water and carbon dioxide between plants and the environment. Maintaining the natural rate of gas exchange and, thus, the rate of photosynthesis is among the important characteristics that play a role in high yields in drylands [11].

Molecular markers are a specific DNA sequence, and their inheritance is easily visible. The use of molecular markers is based on natural polymorphisms in DNA. The first DNA markers are restriction fragment length polymorphisms (RFLPs). A molecular marker must have several desirable characteristics including polymorphism, inheritability, uniform and frequent distribution throughout the genome, affordability of detection, reproducibility, accessibility, and environmentally insensitivity. Unfortunately, no molecular marker has all of these properties. A wide range of molecular techniques reveal polymorphisms at the DNA level. The molecular markers include simple sequence repeats (SSRs), intersimple sequence repeats (ISSRs), inter-retrotransposon amplified polymorphisms (IRAPs), CAAT box-derived polymorphisms (CAATs), start codon targeted polymorphisms (SCoTs), and inter-primer binding sites (iPBSs) [12,13,14,15].

Population and QTL mappings were developed using molecular marker technology. Simultaneously with the identification of QTLs, the breeding of plants was facilitated to withstand abiotic stresses [16,17], and QTL mapping is a useful and accessible tool for plant breeders to clarify the genetic basis of traits related to stress tolerance [18].

Discovering the molecular basis of drought and salinity tolerance in barley seedlings can help develop stress-tolerant genotypes. There are many studies on the genetic control of drought and salinity tolerance in barley. In this regard, QTLs related to root characteristics have been identified [19,20,21]. The QTLs related to leaf length, physiological parameters, osmotic regulation, proline accumulation, relative water content, and leaf wilting have also been identified [22,23,24,25,26,27,28,29,30,31].

However, several studies have examined the effect of drought stress on barley seedlings. Salinity and drought stresses reduce plant leaf area, number, root weight and volume, number, and leaf weight [32,33,34,35,36,37,38], but little attention has been paid to identifying QTLs related to barley seedling traits in drought and salinity stresses. Few studies have been performed to identify the genetic structure of drought tolerance at germination and seedling vigor [7,8,9].

In the study of QTLs controlling germination and early seedling growth using 90 double haploid spring wheat, a total of 38 QTLs were identified [39]. Eighteen loci consisting of QTL phonological traits were mapped to a population-caused BCD47 and Baronesse barley cross [40]. Shahraki et al. [41] mapped QTLs related to phenological traits using 72 double haploid barley lines caused by Strepto and Morx cross under salinity stress.

To identify the position of QTLs in controlling salt tolerance, Ahmadi-Ochtapeh et al. [42] evaluated 162 F8 families resulting from crossing two cultivars: Igri × Arigashar. Four important characteristics, including shoot, root, coleoptile growth, and the number of roots, were strongly influenced by salinity stress during seedling growth stages. Linkage maps were prepared using 106 AFLP and SSR markers. Out of 26 identified QTLs, 17 were identified on chromosomes 2, 3, 4, 6, and 7 for salinity tolerance at 250 and 350 mM NaCl. Considering overlapping QTLs, the pleiotropic effects of nine QTLs were detected including QClgH2.1b, QSdgH2.1b, QSlgH2.1c, QNrgH2.1b, QTwgH2.2c, QSdg3Hb, QSlg4Hb1, QClg4Hb, and QSlg6Hc2. Chromosomes 2 (2H), 4 (4H), and 6 (6H) contained QTLs that were involved in salinity tolerance and significantly increased salinity tolerance in the population. A QTL (QTwg4Hc) on chromosome 4H at distance marker XE41-M61 controlled several features including shoot and coleoptile length.

Liu et al. [43] identified 11 important QTLs distributed across all seven barley chromosomes except chromosome 5, explaining 17.3% of the phenotypic variation. The QTL loci for biomass, intercellular carbon dioxide concentration, transpiration rate, and stomatal conductance were common under control conditions. In addition, QTLs for salinity tolerance were simultaneously identified on chromosome 3 with QTLs for grain yield and biomass. Moreover, candidate genes were proposed for salinity tolerance at this locus. In this study, the absence of major-effect QTLs for gas exchange and stomatal traits under normal and salinity conditions indicated a complex relationship between salinity and gas exchange among quantitative characteristics under multiple gene control.

A challenge for barley breeders is the lack of sufficient information regarding the genes controlling salinity and drought-tolerance traits. The mapping of genes controlling quantitative traits is one of the methods used to study quantitative genetic traits. By identifying the genomic regions controlling quantitative traits and determining the contribution of each region in creating the observed diversity of traits in the population, the efficiency of breeding programs increases, and the modification of populations can be carried out with more confidence [44,45,46,47]. This study aimed to investigate the QTLs controlling the physiological and morphological traits of barley seedlings under normal, salinity, and drought stress conditions in the F8 population resulting from the cross of Badia and Kavir barley cultivars.

## 2. Materials and Methods

### 2.1. Phenotypic Evaluations and Stress Application

In order to locate the genes controlling the physiological and morphological traits in barley, 106 F8 RILs caused by the cross of the Badia and Kavir barley cultivars were studied in a complementary randomized design with three replications under normal, salinity, and drought stress conditions in 2019. This experiment was performed in a growth chamber at the botanical laboratory of Gonbad Kavous University. The cross of the Badia and Kavir barley cultivars had higher and lower performances under normal lodging and stress conditions, respectively [48].

Matured seeds with uniform sizes were selected and cultured in pots 15 cm in diameter and 16 cm in height. The pots had a hole in the bottom for excessive water drainage. The seeds were surface sterilized with 0.1% HgCl_2_ solution for 5 min and thoroughly washed with sterilized distilled water. Ten seeds were planted in each pot and then covered with 1 cm of soil. All plants were grown with 12 h daylight at 28 °C, 12 h dark period at 20 °C, light intensity of 200 μmol.m^−2^.s^−1^, and approximately 70% relative humidity. Moreover, some of the soil’s physical and chemical properties were recorded through extraction with ammonium acetate. The properties included soil texture extracted using the hydrometric method [49], pH and EC using saturated extract method [50], the percentage of carbon with organic carbon using the Walkley and Black method [51], equivalent calcium carbonate with the neutralization of hydrochloric acid [52], the total nitrogen in the soil employing the digestion method [53], the phosphorus extractable with 0.5 M sodium bicarbonate using the Olsen method [54], and absorption of potassium extracted with normal ammonium acetate (Table 1). Watering and seedling management were conducted regularly. When the seedlings were approximately 7 cm tall, each pot was thinned to only 5 seedlings of relatively consistent size with reasonable spacing.

### 2.2. Applying Salinity Stress

After 14 days of growth in the non-saline soil, salinity stress was applied using saline water with a NaCl source at EC 8 dS m^–1^. Salinity stress was raised to EC 16 dS m^–1^ after 7 days of growth at EC 8 dS m^–1^ to prevent plants from any sudden shock. The salinity of the saturated extract was measured on a weekly basis in the degraded pots. Approximately 100 g of potting soil was initially poured into a small plastic bucket to prepare the saturated extract. Subsequently, 65 cc of distilled water was added. It was mixed with a spatula to form a paste or saturated paste. After preparing the saturated mud, we put filter paper on a Buchner’s funnel, poured the saturated paste on it, and extracted the paste with a vacuum pump. A control set was kept without any addition of salt. The instructions for the salinity tolerance are presented in Table 2.

### 2.3. Applying Drought Stress

The pots were filled with 2.2 kg of sterilized field soil containing approximately 6% water. The field capacity, wilting point, and available water content (*AWC*) of the soil were measured at the Gonbad Kavous University’s soil laboratory. To measure the moisture content of the agricultural capacity of the soil, a pot was completely saturated for the sample. In order to prevent surface evaporation of water from the soil, the desired soil was covered with plastic, and the moisture content of the field capacity was measured using the method of preparing a soil sample from a depth of 10 cm and drying it in an oven until it remained constant. The wilting point moisture was obtained by placing the soil sample in a pressure plate device at a suction of 15 atmospheres [55]. Finally, the available water capacity was obtained using the following equation:

(1) $$AWC=\left(\frac{\theta_{PC-}\theta_{PWP}}{100}\right)\times D\times \frac{\rho_{b}}{\rho_{W}}$$

where AWC is the available water capacity; $\theta_{PC}\;$ and $\theta_{PWP}\;$ are, respectively, the weighted moisture content of the field capacity and wilting point; D is the soil depth in centimeters; $\rho_{b}\;$ and $\rho_{W}\;$ are the bulk density of soil and water in g/cm^3^, respectively [56].

Seventy and ten percent *AWC* in the soil were considered for the experiment as the well-watered and severe drought conditions, respectively [57]. The drought treatment was induced after 14 days. The soil moisture for the pots of well-watered and drought stress conditions was maintained with the required amount of water by weighing the pots and irrigating the plants every day. After 14 days, the water in the pots was completely drained using the holes under the pots; after 6 days, the amount of available water decreased to 10% of the *AWC*. By weighing the pots and estimating the amount of water loss, soil dryness remained constant at 10% *AWC* of the available water until harvest. Instructions for determining the drought tolerance are presented in Table 3.

Plant length (PL), plant weight (PW), root weight (RW), root number (RN), root length (RL), leaf weight (LW), leaf length (LI), leaf number (LN), leaf width (LW), chlorophyll content (CHI), stomata length (SL), stomata width (SW), and stomata number (SN) were measured. A genotypic score (SCR) under drought and salinity stress conditions was recorded according to Loresto and Chang [58] (Table 2 and Table 3). Three leaves were randomly selected from each line, and the amount of chlorophyll was read after stress at three points of each leaf using a SPAD chlorophyll meter (SPAD 502 Minolta, Japan) to measure the chlorophyll content [59].

To measure the stomatal features, stomatal imaging was performed based on the method of Mak et al. [60] and O’carrigan et al. [61] with some modifications. Briefly, the third leaf of each plant was collected and transferred to the laboratory in Petri dishes on paper impregnated with stomata stabilizing solution (i.e., 50 mM KCl, 5 mM Na ± MES, and pH 6.1). The abaxial epidermal strips were then removed and placed on slides using a measuring solution (i.e., 10 mM KCl, 5 mM Ca^2+^ MES, and pH 6.1). Aperture images were taken using a CCD camera (Nikon NIS-F1, Tokyo, Japan) attached to a microscope (Leica Microsystems AG, Solms, Germany). All images were taken by a Nikon NIS Element microscope (Nikon, Tokyo, Japan) and were measured using the ImageTool software; then, the stomatal traits were calculated.

### 2.4. Genotype Evaluations

DNA extraction was performed using the CTAB method [62]. Three hundred and sixty-five SSR markers appropriately distributed in seven chromosomes of barley were selected based on [63,64,65,66,67,68]. These SSR primer pairs were examined for polymorphism between two parents, and polymorphic primers were used to amplify the DNA of each plant from the RIL population. One hundred and fifty-two SSR polymorphism markers were used to prepare the primary map. For SSR markers, 50 ng DNA, 0.67 µL primers, 10 µL reaction buffer, 2.5 mM MgCl_2_, 0.2 MM dNTP, and 5.0 units of Taq DNA polymerase (SINACLON Co.) were mixed, and a 15 μL mixture was obtained by adding enough double-distilled deionized water.

The polymerase chain reaction was performed in a thermocycler (Biorad iCycler, USA), and the amplification program included denaturation at 94 °C for 5 min, 30 cycles of denaturation at 94 °C for 1 min, an annealing temperature of 58 °C for 1 min, elongation at 72 °C for 1.5 min, and a final extension at 72 °C for 5 min. The amplified products were electrophorized on a 6% polyacrylamide gel electrophoresis (PAGE) and made visible by silver staining [69]. For the primary saturation map, the used markers included: iPBS [70], IRAP [71,72], ISSR (University of British Columbia (UBC)), SCoT [73], and CAAT [74]. Twenty-one IPBS markers, eight IRAP markers, fifteen SCoT markers, fifteen CAAT markers, and a combination of ISSR and iPBS markers were used to evaluate the polymorphisms of the above markers in parents. Finally, 7 IRAP alleles, 29 CAAT alleles, 27 SCoT alleles, 72 ISSR alleles, 15 IPBS alleles, and five combinations of ISSR and iPBS polymorphic alleles were used to generate genetic maps. For genetic mapping, scores 1 and 2 were used for the male and female parent bands in the SSR markers, respectively. For markers. For per chromosomes, the binding of random markers (i.e., ISSR, iPBS, IRAP, SCoT, and CAAT) to microsatellite markers was carried out separately. Polymorphic bands for each of the iPBS, IRAP, ISSR, SCoT, and CAAT primers were numbered in descending order (from the top to the bottom of the gel) of molecular weight.

### 2.5. Linkage Map Construction and QTL Analysis

All iPBS, IRAP, ISSR, SCoT, CAAT, and SSR polymorphic alleles were evaluated separately using the χ2 test for a segregation ratio of 1:1 at a probability level of 0.01, and those without a 1:1 ratio were removed from the genotypic data. Map preparation was accomplished using Map Manager QTX17 [75]. Assignment of linkage groups to relevant chromosomes was performed based on [63,64,65,66,67,68]. Map distances were based on the Kosambi function, and the critical logarithm of odd (LOD) threshold of 2.5 was used to determine the associating groups. QGENE software [76] was used to find QTLs; the composite interval mapping (CIM) method was used to determine the QTLs and estimate their effects, and the point with the highest was identified as the QTL region with the highest LOD probability.

## 3. Results

### 3.1. Phenotypic Distribution and Relationships between Traits

Our phenotypic study on the traits suggested the presence of quantitative and continuous variations. Among the studied lines, individuals were identified whose trait values were higher or lower than the parents, which indicates transggressive segregation of all traits.

The difference between normal and drought stress conditions was significant for all traits (Table 4). In addition, the difference between normal and salinity stress conditions was significant for all traits. All traits decreased due to the presence of drought and salinity stresses, except SCR which increased due to the stress.

**Figure 1:**
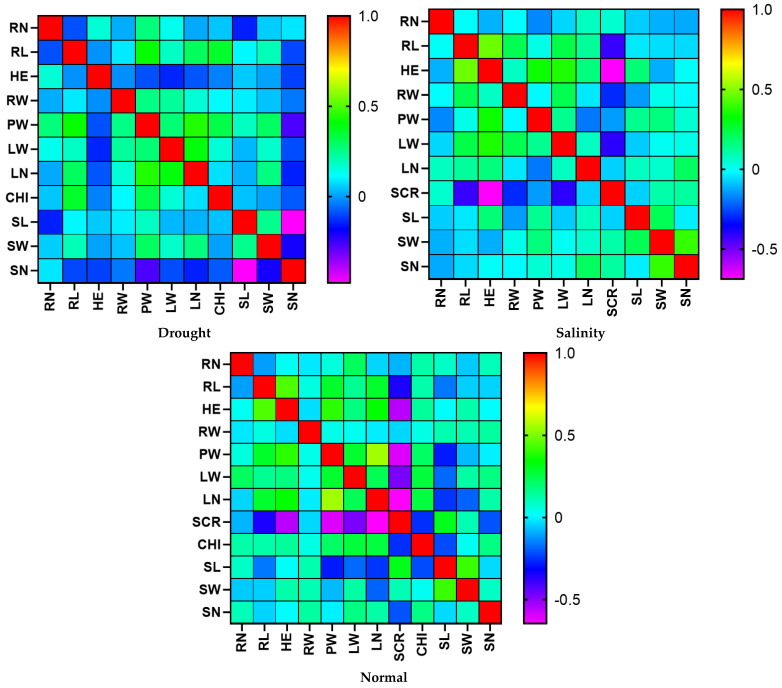
Correlation diagram of the physiological and morphological traits under salinity, drought, and normal stress in the seedling stage of the RIL population caused by the Kavir and Badia cross. The red to yellow color spectrum indicates a correlation between 1 and 0.5. Next, the green color spectrum indicates a correlation between 0.5 and 0. The light blue spectrum indicates a correlation between 0 and −0.5. Finally, the dark blue to navy blue color spectrum indicates a correlation between −0.5 and −1.

The PW had significant positive correlations with LW, LN, CHI, and SW, but it had a significant negative correlation with SN in normal conditions (Figure 1). However, PW was significantly correlated with LL in drought stress. The SCR showed significant negative correlations with RL, LL, RW, and LW. In salinity stress conditions, PW had significant positive correlations with LW, LN, and CHI but showed significant negative correlations with SCR and SL. The SCR showed significant negative correlations with RL, LL, PW, LW, and LN.

The results showed that the PW in both normal and salinity conditions significantly positively correlated with LW, LN, and CHI. In addition, SCR significantly correlated with RL, LL, and LW under drought and salinity stress conditions.

Stepwise regression was used to select traits with a more critical role in explaining the variation in normal, drought, and salinity stress conditions. The results of the forward regression in normal conditions showed that when PW was considered as a dependent variable and other traits as independent variables, the LN, RL, RN, SN, and CHI content entered into the model and explained 44.1% of the PW variation (Table 5). The results of the stepwise regression under drought stress conditions showed that when PW was considered as a dependent variable and other traits as independent variables, LI, LN, and SW fed into the model explained 11, 17.5, and 22.3% of the PW variation, respectively. For the selection of the traits with a more significant effect on the SCR in drought stress conditions, the results of the stepwise regression showed that LI, RW, and LW explained 50.7, 57.4, and 59.2% of the SCR variation, respectively (Table 6). In addition, under salinity stress, when PW was considered as a dependent variable and other traits as independent variables, SCR and LN fed into the model explained 40.5% of PW variation. Similarly, when SCR was considered as a dependent variable and other traits as independent variables, LN, LW, PW, and SL explained 65% of the variation of SCR (Table 7).

**Figure 2:**
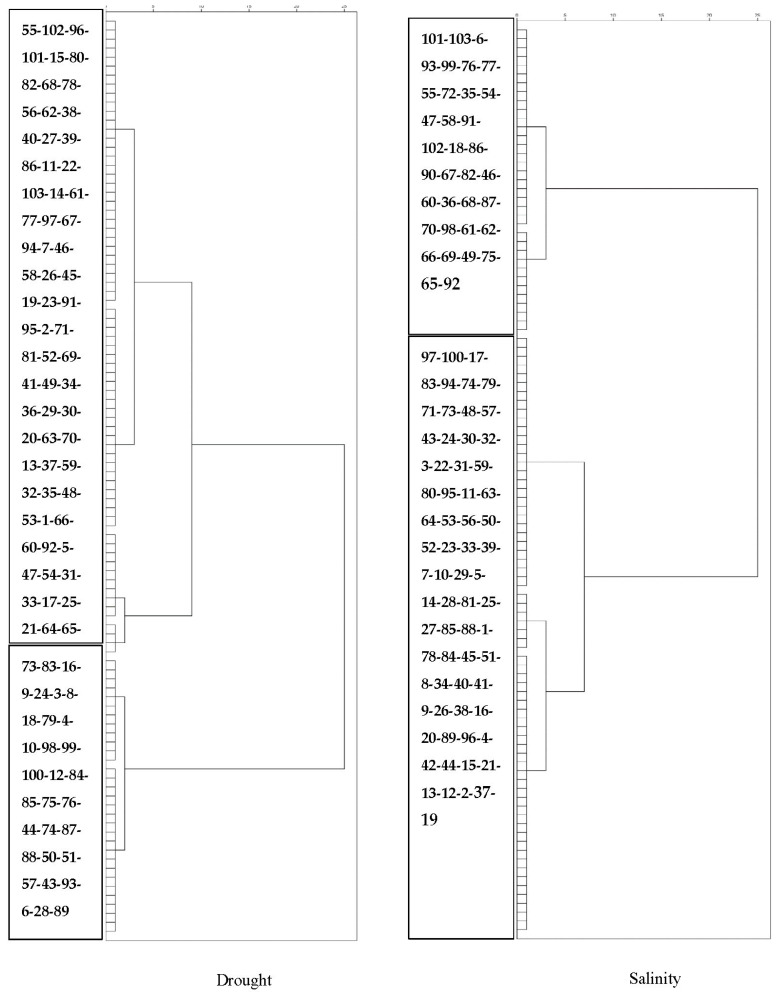
Dendrogram based on the genetic code under salinity and drought stress conditions.

Cluster analyses divided barley lines into two groups: drought and salinity stress conditions (Figure 2). The first group had 71 genotypes and an average SCR of 5.04 (sensitive and relatively sensitive), and the second group had 32 genotypes and an average SCR of 2.81 (tolerant and relatively tolerant). In salinity stress, the first group had 35 genotypes and an average SCR of 5.44 (sensitive and relatively sensitive), and the second group had 68 genotypes and an average SCR of 3.97 (tolerant and relatively tolerant).

### 3.2. Preparation of Linkage Maps

**Figure 3:**
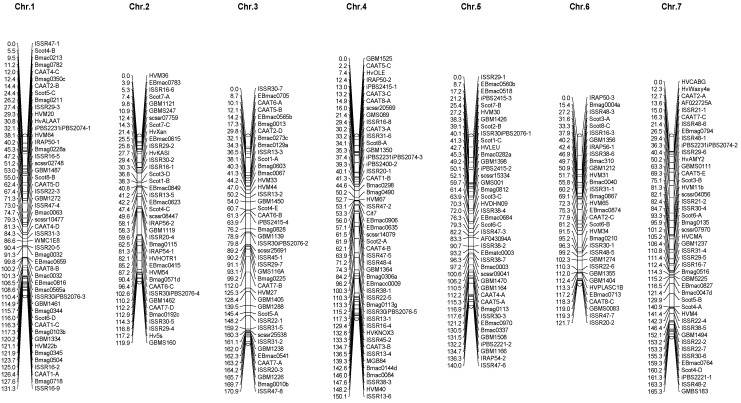
Linkage maps of barley chromosomes based on SSR, ISSR, IRAP, iPBS, CAAT, and SCoT markers in the RIL population caused by the Kavir and Badia crosses.

Linkage maps were prepared using 152 SSR markers, 72 ISSR alleles, 7 IRAP alleles, 29 CAAT alleles, 27 SCoT alleles, and 15 iPBS alleles (Figure 3). The mendelian ratio of 1:1 for all amplified alleles was evaluated using the chi-square test, and non-Mendelian transgressive segregate markers were not involved in map construction. The molecular markers were assigned to seven linkage groups on seven barley chromosomes. Chromosome 1 had the highest number of markers (50) and a contracting length of 131.3 cM. In addition, 39, 44, 49, 42, 33, and 45 markers were assigned to chromosomes 2, 3, 4, 5, 6, and 7, respectively. Their lengths were 190.9, 170.9, 150.1, 140.1, 121.7, and 165.3 cM, respectively (Figure 3). The arrangement of SSR markers was different from the maps in [44,45,46,49], but their chromosomal locations matched their maps.

The first linkage map of barley was prepared by Graner et al. [77] using RFLP markers, and 71 double haploid populations caused by Igri × Franka. In addition, linkage maps of barley were prepared by following the doubled haploid population caused by the Steptoe and Morex crosses. Moreover, Kleinhofs et al. [78] (RFLP marker), Wenzl et al. [79] (SSR, RFLP, STS, and DArT markers), Qi et al. [80] (AFLP, RFLP, and SST markers), Jafary et al. [81] (AFLP and SSR markers), Thiel et al. [68] (SSR and EST markers), Sato et al. [82] (SNP marker), Varshney et al. [83] (RFLP, SNP, and SSR markers), and Rostoks et al. [84] (SNP and SSR markers) prepared other barley genetic linkage maps.

### 3.3. Mapping Quantitative Traits under Normal Condition

For the traits evaluated under normal conditions, eight QTLs were identified, responsible for controlling seven traits (Table 8). qSNN-3 was identified for the SN on chromosome 3 with an LOD = 2.64, and it was located at 44 cM between the Bmac0067 and HVM33 markers. qSNN-3 explained 11.2% of the phenotypic variation in the trait and acted to reduce SN with an additive effect of −2.319. The reducing alleles were transferred from the parent Badia to the offspring.

Two QTLs (i.e., qRLN-7a and qRLN-7b) were identified for RL on chromosome 7 in 66 and 134 cM, with and of LOD = 2.53 and LOD = 2.60, respectively. They were located between GBMS0111 and CAAT5-E, as well as the SCoT5-B and SCoT4-A markers. qRLN-7a and qRLN-7b explained 10.8 and 11.1% of the phenotypic variation in RL, respectively. The additive effect in the first position was equal to −1.603 in the decreasing direction, and the reducing alleles were transferred from the parent Badia to the offspring. However, in the second position, the additive effect was 1.98, and the increasing alleles transferred from the parent Kavir to the offspring.

For LI, two QTLs (i.e., qLIN-2 and qLIN-4) were identified on chromosomes 2 and 4 at 60 and 70 cM, respectively, between ISSR20-4 and Bmag0115, as well as between ISSR47-5 and ISSR48-4. The QTLs qLIN-2 and qLIN-4 explained 11.1 and 11.9% of phenotypic variation in LI, respectively. These reducing alleles were transferred from the parent Badia to the offspring.

qLWN-2 for LW was identified on chromosome 2 at 14 cM near the scssr07759 and SCoT7-C. This QTL explained 12% of the LW phenotypic variation. It reduced LW with the additive effect of −2.19, and the parent Badia alleles reduced this trait.

qLNN-1 and qCHN-3 controlled LN and CHI on chromosomes 1 and 3, explaining 11% of the phenotypic variation. qLNN-1 had an additive effect of 0.862, and the Kavir parent alleles increased, but in qCHN-3, the parent Badia alleles decreased (−0.221) CHI.

### 3.4. Mapping of Quantitative Traits under Drought Stress Condition

Nine QTLs were identified under drought stress, responsible for controlling up to seven traits. For SN, qSND-1 was located on chromosome 1 at 58 cM. qSND-1 was considered as a major-effect QTL with an LOD = 3.874 between the SCoT8-B and CAAT5-D markers, explaining 16.5% of the total phenotypic variation by increasing the parent Kavir alleles in the offspring (Table 9).

qRWD-2 was detected for RW on chromosome 2 at 90 cM between HVM54 and Bmag0571, with an additive effect of 0.017. qRWD-2 acted in the increasing direction and was transferred from the parent Kavir to the offspring. This QTL explained 14.5% of the RW phenotypic variation.

For LW, two QTLs (i.e., qLWD-1 and qLWD-7) were identified on chromosomes 1 and 7. The QTL located on chromosome 1 was at 32 cM between HvALAAT and iPBS2231iPBS2074-1, explaining 11.2% of the phenotypic variation in LW. The other QTL (i.e., qLWD-7) was located at 40 cM on chromosome 7 between iPBS2231iPBS2074-2 and ISSR29-6, explaining 12.1% of the phenotypic variation in LW. Unlike qLWD-1, it had an increasing effect of 0.05, and the parent Kavir alleles increased this trait.

A major-effect QTL (i.e., qLWD-2) for LW was detected on chromosome 2 at 108 cM, located between ISSR30iPBS2076-4 and GBM1462, explaining 15.2% of the LW phenotypic variation. In addition, increasing alleles were transferred from the parent Kavir (0.27) to the offspring.

For LN, qLND-4 and qLND-5 were identified on chromosomes 4 and 5. qLND-4 on chromosome 4, at 136 cM between the CAAT3-B and ISSR13-4 markers, had an LOD = 2.95, explaining 12.5% of the phenotypic variation in LN. The QTL (i.e., qLND-4) additive effect was −0.203 in the reducing direction, and parent Badia alleles reduced this trait. qLND-5 on chromosome 5 was at 82 cM, between SCoT6-C and ISSR47-3, explaining 13.8% of the phenotypic variation in leaf number. Its additive effect was 0.248 in the increasing direction, and the parent Kavir alleles transferred this trait.

For SCR, two QTLs (i.e., qSCD-3 and qSCD-7) were identified on chromosomes 3 and 7 at 16 and 38 cM, and the LODs were 2.59 and 2.54, respectively. They were located between EBmac0565 and Bmag0013 and iPBS2231iPBS2074-2 and ISSR29-6, explaining 11.1% and 10.9% of the phenotypic variation in SCR, respectively. Both qSCD-3 and qSCD-7 were in the reducing direction and transferred from the parent Badia alleles to the offspring.

### 3.5. Mapping of the Quantitative Traits under Salinity Stress Conditions

For the traits evaluated under salinity stress conditions, 26 QTLs controlling six traits were identified. Six QTLs were identified on chromosomes 1 (qSLS-1a and qSLS-1b), 4 (qSLS-4), 5 (qSLS-5), 6 (qSLS-6), and 7 (qSLS-7) for SL. qSLS-1a and qSLS-1b were at 28 and 108 cM with LODs of 3.31 and 2.69, respectively. They were located between ISSR29-3 and HVM20 as well as EBmac0816 and Bmac0565, explaining 14 and 11.5% of the phenotypic variation, respectively. A major-effect QTL (i.e., qSLS-4) was identified on chromosome 4 at 58 cM. Its LOD was 5.145, between EBmac0635 and scssr14079, and it explained 20.9% of the phenotypic variation in SL. qSLS-5 was detected on chromosome 5 at 94 cM and between EBmatc0003 and ISSR38-7, which explained 11.6% of the phenotypic variation in SL. On chromosome 6, a QTL was detected for SL at position 0 (LOD = 2.88). It was located next to IRAP50-3, explaining 12.3% of the phenotypic variation in SL. The last QTL for SL (qSLS-7) was found on chromosome 7 at 100 cM with an LOD = 3.652. It was located between Bmag0135 and scssr07970, explaining 15.3% of the phenotypic variation in SL (Table 10).

The qSLS-1a, qSLS-1b, qSLS-4, qSLS-5, and qSLS-7 additive effects were −2.692, −2.212, −0.568, −1.918, and −2.298, respectively. The reducing alleles were transferred from the parent Badia to the offspring. However, qSLS-6 had an increasing effect of 3.339, and the parent Kavir alleles increased this trait.

For the SW, the qSWS-4 with an LOD = 2.863 was identified at 28 cM on chromosome 4, between GMS089 and ISSR16-8, explaining 12.2% of the phenotypic variation in SW. The qSWS-4 additive effect was −1.502 in the reducing direction, and the parent Badia alleles reduced this trait.

For RL, two QTLs were identified on chromosomes 1 (qRLS-1) and 4 (qRLS-4) at 126 and 140 cM with LODs of 2.72 and 2.84, respectively. They were located between the ISSR16-2 and CAAT1-A and MGB84 and Bmac0144 markers, respectively. qRLS-1 and qRLS-4 explained 11.5 and 11.9% of RL’s phenotypic variations, and with an additive effect of 2.94 and 1.03, respectively, both acted in the increasing direction, and the parent Kavir alleles increased the RL.

For LW, seven QTLs were identified on chromosomes 2 (qLWS-2), 3 (qLWS-3), 4 (qLWS-4a and qLWS-4b), 5 (qLWS-5), 6 (qLWS-6), and 7 (qLWS-7). The QTL on chromosome 2 with an LOD = 2.65 was found at 4 cM, between EBmac0783 and ISSR16-6, explaining 11.2% of the phenotypic variation in LW. The QTL additive effect was −0.029 in the reducing direction, and the parent Badia alleles reduced the leaf weight. The QTL on chromosome 3 with an LOD = 2.95 was identified at 44 cM. It was identified between Bmac0067 and HVM336, explaining 12.4% of the phenotypic variation in LW.

In addition, a major-effect QTL (i.e., qLWS-4a) at 56 cM and a QTL at 140 cM on chromosome 4 were found for LW with LODs of 5.21 and 3.62, respectively. They were located between EBmac0906 and EBmac0635 as well as MGB84 and Bmac0144, explaining 20.8 and 9.14% of the phenotypic variation in LW, respectively. qLWS-5 for LW was detected on chromosome 5 at 94 cM with an LOD = 3.31, between EBmatc0003 and ISSR38-7, explaining 13.8% of the phenotypic variation in LW. The QTL on chromosome 6 with an LOD = 2.754 was detected at 74 cM. qLWS-6 was identified between HVM65 and EBmac0874, explaining 11.6% of the phenotypic variation in LW. qLWS-7 for LW on chromosome 7 was found at 98 cM, between Bmag0135 and scssr07970, explaining 14.4% of the phenotypic variation in LW. The qLWS-3, qLWS-4a, qLWS-4b, qLWS-5, qLWS-6, and qLWS-7 additive effects were 0.03, 0.04, 0.04, 0.03, and 0.03, respectively. They acted in an increasing direction, and the parent Kavir increased LW.

For LN, five QTLs were identified on chromosomes 1 (qLNS-1), 4 (qLNS-4), 6 (LNS-6), and 7 (qLNS-7a and qLNS-7b). The QTLs on chromosomes 1, 4, and 6 were located at 28, 56, and 62 cM, and their LODs were 2.97, 3.91, and 2.7, respectively. They explained 12.5, 1.6, and 11.7% of the phenotypic variation in LN, respectively. These three QTLs acted in the increasing direction, and the parent Kavir alleles increased the LN. Two major-effect QTLs for LN were identified on chromosome 7 at 62 and 98 cM with LODs of 4.05 and 4.468, respectively. They were identified between HvAMY2 and GBMS0111 as well as Bmag0135 and scssr07970. qLNS-7a and qLNS-7b explained 16.6% and 18.1% of the phenotypic variation in LN, respectively. The QTLs additive effect at the first position was −0.14 in the decreasing direction, and the parent Badia alleles reduced this trait. However, the increasing effect of the second position was 0.159 in the increasing direction, and the parent Kavir alleles increased LN.

For SCR under salinity stress conditions, five QTLs were found on chromosomes 1 (qSCS-1), 4 (qSCS-4), 6 (qSCS-6a and qSCS-6b), and 7 (qSCS-7) at 126 (major-effect QTL), 56, 62, 74, and 98 cM (major-effect QTL), respectively, explaining 15.9, 1.2, 11.4, 11.4, and 18.1% of the phenotypic variation in SCR, respectively. The additive effect of these QTLs were −1.276, −0.317, −0.51, −0.383, and −0.481, respectively, and acted in the reducing direction, and the reducing alleles were transferred from the Badia parent.

## 4. Discussion

The aim of this study was to investigate changes in the emergence of quantitative traits related to QTLs simultaneously under normal, drought, and salinity conditions. Transgressive segregation was observed for all study traits. Many QTL studies have also reported transgressive segregation [85,86]. Such characteristics increase the probability of identifying QTLs and show that both parents contain desirable and undesirable alleles for different traits [85]. This phenomenon can result from recombination, minor QTLs, epistasis, the interaction of genotype with the environment, and mutation during the production of new populations.

Using correlation coefficients between different traits, making more accurate decisions on indirect selection indices, and eliminating ineffective traits are possible [87]. In this study, SCR showed significant negative correlations with RL, LI, PW, LW, and LN, because plants with a better response to drought and salinity stresses (i.e., lower SCR) are more valuable in terms of these traits.

Influential traits in a regression model can be used as suitable selection criteria for increasing PW and, thus, improving drought and salinity tolerance in barley. In this study, when PW was considered as a dependent variable and the other traits as independent variables, LN fed into the model under all three conditions (i.e., normal, drought, and salinity). In addition, leaf length and leaf width traits entered the model under drought and salinity stresses when SCR was considered as a dependent variable and the other traits as independent variables.

Cluster analysis is a powerful tool for selecting genotypes effectively when modifying plants through multiple traits [88]. The present study divided the lines into two groups under normal, drought, and salinity conditions: the sensitive and relatively sensitive lines in both the drought and salinity conditions included lines 101, 77, 55, 72, 35, 54, 47, 58, 91, 102, 86, 67, 82, 46, 60, 36, 68, 70, 61, 62, 69, 49, 65, and 92. The tolerant and relatively tolerant lines under the drought and salinity stress conditions included the lines 73, 83, 16, 9, 24, 79, 4, 10, 100, 12, 84, 85, 44, 74, 88, 50, 51, 57, 43, 28, and 89. Parental selection can be carried out from the tolerant and sensitive clusters in cross-breeding and parent selection programs to obtain drought- and salinity-tolerant lines.

In our study, qSLS-1a for stomatal length and qLNS-1 for LN under salinity stress were simultaneously identified on chromosome 1 at 28 cM and mapped between ISSR29-3 and HVM20. In their studies on rice seedlings under drought stress, Amani Daz et al.’s [89] study on rice seedlings under drought stress reported one QTL for stomatal density and stomatal surface in pre- and post-stress conditions.

qSCS-6a and qLNS-6 were mapped on chromosome 6 at 62 cM in the region between ISSR31-1 and Bmag0867. In addition, QTLs for leaf weight (qLWS-6) and genetic score (qSCS-6b) were collocated on chromosome 6 at 74 cM, between HVM65 and EBmac0874.

Under salinity stress, a large-effect QTL for SCR (qSCS-1) and a QTL for root length (qRLS-1) were found on chromosome 1 at 126 cM, between ISSR16-2 and CAAT1-A, and in the same region, a QTL related to LN was detected under normal conditions. In wheat, Liu et al. [90] identified three QTLs for RL under drought stress conditions on chromosomes 2D, 3A, and 5B. Moreover, in rice, Courtois et al. [91] detected three QTLs for root length in which the source of the VrnH1 marker was linked to a major-effect QTL allele, and RL increased by 9%. Similarly, Chen et al. [92] found a QTL for RL in the 5H chromosome in barley.

Under normal conditions and salinity stress, chromosomes 1, 6, and 3 play a more important role in the salinity tolerance of barley due to the colocation of major-effect QTLs. However, in the present study, the QTL for LL was on chromosome 4 at 70 cM under normal conditions. Taghizadeh et al. [93] found one QTL (qNS-4) on chromosome 4 at 70 cM, which was related to the total spike number and explained 10.5% of the trait variation. In addition, Moslemi et al. [94] detected a QTL for the LI at 32.7 cM on a chromosome 4.

This study identified a QTL for leaf width on chromosome 2 at 14 cM under normal conditions. On the same chromosome and at the same position, Ghaffari Moghadam et al. [95] identified one QTL (qSLn-2a) for stem length, explaining 2.9% of phenotypic variation in the trait.

Under normal conditions, we found a QTL for CHI on chromosome 3 at 164 cM. In a study by Golshani and Fakheri [96], two QTLs for CHI were identified on chromosome 3 at 75.30 and 131.80 cM from ABG398 and CDO113B. In normal conditions, Fakheri and Mehravaran [97] identified three QTL loci on chromosome 2 and one QTL on chromosome 3, explaining 26.49, 21.21, 19.35, and 13.27% of CHI variation, respectively.

We detected a QTL for LN on chromosome 5 at 82 cM. On the same chromosome at 82 cM, Ghaffari Moghadam et al. [95] tracked a QTL (qSLn-5) for seedling length, explaining 12.2% of the phenotypic variation in the trait under drought stress.

This study mapped a QTL for the genetic score on chromosome 3 at a 16 cM under drought stress conditions. Likewise, in a study under normal conditions, Moslemi et al. [94] reported a QTL for root fresh weight on chromosome 3 at 16.3 cM, explaining 12.7% of the changes in the SCR.

Arifuzzaman et al. [98] found three QTLs for RL in barley seedlings on chromosomes 2H, 3H, and 5H. They mapped these QTLs (i.e., QRl.S42.2H, QRr.S42.3H, and QRl.S42.5H) at 141.1, 118.72, and 125.1 cM, which contrasts with the findings of the present study.

Mapping of QTLs for barley characteristics was examined by Siahsar and Narouei [99] and Arifuzzaman et al. [81] at the seedling stage under salinity stress and by Mano and Takeda [100] at the germination stage. Xue et al. [101] investigated a major-effect QTL on chromosome 7 in the Nure × Tremois population of barley seedlings in response to salinity stress. Wang et al. [102] evaluated 21 characteristics of barley seedlings under hydroponic conditions. The characteristics were related to leaf age (LAG), branch height (SH), maximum root length (MRL), main root number (MRN), and seedling fresh weight (SFW).

## 5. Conclusions

This study aimed to identify the main characteristics of seedlings under hydroponic condition and to compare patterns of QTL expression among four different seedling growth stages. We report the first study regarding QTL on an Iranian barley population under normal, salinity, and drought stress conditions. For the traits evaluated under normal, drought, and salinity conditions, 8, 9, and 26 QTLs were identified. Identification of QTLs with positive and negative incremental effects indicated the transfer of desirable alleles at these loci from both parents to the offspring, and both parents, Badia and Kavir, can be used in barley breeding programs. We identified several QTLs (i.e., qSCS-1, qRLS-1, qLNN-1, qLWS-4a, qSLS-4, qLNS-7b, qSCS-7, and qLNS-7a) for SCR, LN, LW and SL with higher expression of phenotypic variation in the studied traits, they were considered as major-effect QTLs, and markers associated with them can be used in breeding programs to select families and transfer alleles.

## Figures and Tables

**Table 1:** Soil physical and chemical properties of the experiment site (0–30 cm depth).

Sand	Silt	Clay	Potassium	Phosphorus	N	Organic Carbon	Neutral Substances	pH	EC
(%)	(%)	(%)	(ppm)	(ppm)	(%)	(%)	(%)		(ds/m)
13	58	29	316	11.4	0.09	0.90	9.5	7.6	1.19

**Table 2:** Instructions for determining the response to salinity stress.

Reaction	Symptoms	Genetic Score
Highly Tolerant	Normal growth, no leaf symptoms	1
Tolerant	Nearly normal growth but leaf tips or a few leaves are whitish and rolled	3
Moderately Tolerant	Growth severely retarded, most leaves rolled, and only a few are elongated	5
Sensitive	Complete cessation of growth, most leaves are dry, and some plants are dying	7
Highly Sensitive	Almost all plants are dead or dying	9

**Table 3:** Instructions for determining the response to drought stress.

Reaction	Leaf Rolling	Leaf Burning	Genetic Score
Highly Tolerant	No signs of stress	No signs of stress	0
Tolerant	No leaf rolling	Partial burning of leaf tips	1
Moderately Tolerant	Partially rolling and no rolling in the evening	Dissipation of leaf tip burning by a quarter of the leaves	3
Moderately Sensitive	Partially; no rolling at late evening and early morning	Burning of half of the young leaves and all of the lower leaves	5
Sensitive	Fully rolling and no rolling in the morning	Burning of the leaves spread to three-quarters of the leaves	7
Highly Sensitive	Like tube and rolling in the morning	Burning spread to all of the leaves	9

**Table 4:** Means, standard errors, and *t*-test between normal and stresses conditions for traits.

Traits	Mean ± Standard Error			*t* Statistic	
	Normal	Drought	Salinity	*t* Normal-Drought	*t* Normal-Salinity
RN (no.)	5.81 ± 0.04	5.20 ± 0.038	5.28 ± 0.041	11.04 **	9.12 **
RL (cm)	16.12 ± 0.24	13.13 ± 0.282	15.41 ± 0.241	8.07 **	2.08 **
HE (cm)	50.27 ± 19.43	13.11 ± 0.358	18.37 ± 0.251	1.26 **	1.09 **
RW (gr)	0.092 ± 0.01	0.061 ± 0.003	0.08 ± 0.009	3.72 **	0.82 **
PW (gr)	0.47 ± 0.01	0.10 ± 0.007	0.19 ± 0.008	26.22 **	19.56 **
LW (gr)	2.34 ± 0.07	1.80 ± 0.050	2.42 ± 0.058	6.29 **	−0.88 **
LN (no.)	2.11 ± 0.07	2.87 ± 0.027	1.36 ± 0.28	−10.04 **	10.04 **
Ll (cm)	32.35.15 ± 0.09	19.58 ± 0.031	17.24 ± 0.31	−7.02 **	4.01 **
SCR	0.93 ± 0.02	4.39 ± 0.121	4.51 ± 0.09	−28.16 **	−40.15 **
SL (µm)	41.58 ± 0.35	36.32 ± 0.385	37.13 ± 0.47	10.14 **	7.59 **
SW (µm)	21.76 ± 0.22	17.63 ± 0.230	17.46 ± 0.21	13.07 **	14.10 **
SN (no.)	37.87 ± 0.64	25.92 ± 0.667	35.82 ± 0.55	12.95 **	2.42 **

** Significance at a 1% probability level.

**Table 5:** Forward regression under normal conditions when plant weight was considered as a dependent variable and the other attributes as independent variables.

Variable	Regression Coefficient	Mean of Squares	F	R^2^
LN	0.05 **	0.30	24.51	0.2
RL	0.01 **	0.21	19.58	0.28
RN	0.08 **	0.19	19.03	0.37
SN	−0.00 **	0.16	17.17	0.45
CHI	0.07 *	0.13	15.15	0.44

* and ** represent significant in 5 and 1% probability levels.

**Table 6:** Forward regression in drought conditions for when plant weight and genetic score were considered as dependent variables and the other attributes as independent variables.

Variable	Regression Coefficient	Mean of Squares	F	R^2^
Plant Weight				
LI	0.01 **	0.57	11.99	11.0
LN	−0.08 **	0.45	10.19	17.5
SW	0.01 *	0.38	9.11	22.3
**Genetic Score**				
LI	−0.22 **	79.26	99.75	50.7
RW	−9.95 **	44.86	64.64	57.4
LW	−0.36 *	30.85	45.97	59.2

* and ** represent significant in 5 and 1% probability levels.

**Table 7:** Forward regression in salinity stress conditions for when plant weight and genetic code were considered as dependent variables and the other attributes as independent variables.

Variable	Regression Coefficient	Mean of Squares	F	R^2^
Plant Weight				
SCR	−0.04 **	0.23	56.12	36.2
LN	0.07 **	0.13	33.37	40.5
**Genetic Score**				
LN	−1.01 **	29.73	68.41	40.9
PL	−0.11 **	19.48	56.48	53.5
LW	−0.37 **	15.16	53.93	62.5
LI	−1.96 *	11.89	45.33	65.4
SL	0.22 *	9.72	38.23	65.9

* and ** represent significant in 5 and 1% probability levels.

**Table 8:** QTLs controlling traits in barley under normal conditions in seedling in the RIL population caused by the Kavir and Badia crosses.

Traits	QTL	Chr	Position	Flanking Markers	Distance to Closer Marker	LOD	Add Effect	R^2^	Allele Direction
SN	qSNN-3	3	44	Bmac0067-HVM33	0.23 (HVM33)	2.64	−2.32	11.2	Badia
RL	qRLN-7a	7	66	GBMS0111-CAAT5-E	2.75 (GBMS0111)	2.53	−1.60	10.8	Badia
	qRLN-7b	7	134	SCoT5-B- ScoT4-A	4.13 (ScoT5-B)	2.60	1.98	11.1	Kavir
LI	qLIN-2	2	60	ISSR20-4-Bmag0115	0.39 (ISSR20-4)	2.61	−2.14	11.1	Badia
	qLIN-4	4	70	ISSR47-5-ISSR48-4	1.15 (ISSR48-4)	2.80	−2.21	11.9	Badia
LW	qLWN-2	2	14	scssr07759-Scot7-C	0.26 (ScoT7-C)	2.84	−2.19	12	Badia
LN	qLNN-1	1	126	ISSR16-2-CAAT1-A	0.41 (CAAT1-A)	2.50	0.86	10.7	Kavir
CHI	qCHN-3	3	164	CAAT7-A-ISSR20-3	0.23 (ISSR20-3)	2.62	−0.22	11.2	Badia

**Table 9:** QTLs controlling traits in barley under normal conditions at the seedling stage in an RIL population caused Kavir and Badia crosses.

Traits	QTL	Chromosome	Position	Flanking Markers	Distance to Closer Marker	LOD	Add Effect	R^2^	Allele Direction
SN	qSND-1	1	58	SCoT8-B- CAAT5-D	3.04 (SCoT 8-B)	3.87	6.10	16.5	Kavir
RW	qRWD-2	2	90	HVM54- Bmag0571	0.36 (Bmag0571)	3.48	0.02	14.5	Kavir
LW	qLWD-1	1	32	HvALAAT-iPBS2231iPBS2074-1	0.13 (iPBS2231iPBS2074-1)	2.64	−0.35	11.2	Badia
	qLWD-7	7	40	iPBS2231iPBS2074-2-ISSR29-6	0.36 (ISSR29-6)	2.87	0.05	12.1	Kavir
	qLWD-2	2	108	ISSR30iPBS2076-4-GBM1462	2.23 (GBM1462)	3.65	0.27	15.2	Kavir
LN	qLND-4	4	136	CAAT3-B- ISSR13-4	0.45 (ISSR13-4)	2.95	−0.20	12.5	Badia
	qLND-5	5	82	SCoT6-C- ISSR47-3	0.16 (ISSR47-3)	3.29	0.25	13.8	Kavir
SCR	qSCD-3	3	16	EBmac0565-Bmag0013	1.26 (Bmag0013)	2.59	−0.56	11.1	Badia
	qSCD-7	7	38	iPBS2231iPBS2074-2-ISSR29-6	1.75 (iPBS2231iPBS2074-2)	2.54	−0.95	10.9	Badia

**Table 10:** QTLs controlling traits in barley under salinity stress at the seedling stage in the RIL population caused the Kavir and Badia crosses.

Traits	QTL	Chromosome	Position	Flanking Markers	Distance to Closer Marker	LOD	Add Effect	R^2^	Allele Direction
SL	qSLS-1a	1	28	ISSR29-3- HVM20	0.74 (ISSR29-3)	3.31	−2.69	14	Badia
	qSLS-1b	1	108	EBmac0816-Bmac0565	0.62 (Bmac0565)	2.69	−2.21	11.5	Badia
	qSLS-4	4	58	EBmac0635-scssr14079	0.88 (EBmac0635)	5.145	−2.57	20.9	Badia
	qSLS-5	5	94	EBmatc0003- ISSR38-7	0.76 (EBmatc0003)	2.69	−1.92	11.6	Badia
	qSLS-6	6	0	IRAP50-3	IRAP50-3	2.88	3.34	12.3	Kavir
	qSLS-7	7	100	Bmag0135- scssr07970	1.23 (scssr07970)	3.65	−2.29	15.3	Badia
SW	qSWS-4	4	28	GMS089-ISSR16-8	1.41 (ISSR16-8)	2.86	−1.50	12.2	Badia
RL	qRLS-1	1	126	ISSR16-2- CAAT1-A	0.41 (CAAT1-A)	2.72	2.94	11.5	Kavir
	qRLS-4	4	140	MGB84- Bmac0144	0.72 (MGB84)	2.84	1.03	11.9	Kavir
LW	qLWS-2	2	4	EBmac0783- ISSR16-6	0.09 (EBmac0783)	2.65	−0.03	11.2	Badia
	qLWS-3	3	44	Bmac0067- HVM33	0.23 (HVM33)	2.95	0.03	12.4	Kavir
	qLWS-4a	4	56	EBmac0906-EBmac0635	0.03 (EBmac0906)	5.21	0.04	20.8	Kavir
	qLWS-4b	4	140	MGB84- Bmac0144	0.72 (MGB84)	3.62	0.04	14.9	Kavir
	qLWS-5	5	94	EBmatc0003- ISSR38-7	0.76 (EBmatc0003)	3.31	0.03	13.8	Kavir
	qLWS-6	6	74	HVM65- EBmac0874	1.26 (EBmac0874)	2.75	0.03	11.6	Kavir
	qLWS-7	7	98	Bmag0135- scssr07970	2.25 (Bmag0135)	3.48	0.039	14.4	Kavir
LN	qLNS-1	1	28	ISSR29-3- HVM20	0.74 (ISSR29-3)	2.97	0.15	12.5	Kavir
	qLNS-4	4	56	EBmac0906-EBmac0635	0.03 (EBmac0906)	3.91	0.12	1.6	Kavir
	qLNS-6	6	62	ISSR31-1- Bmag0867	1.77 (ISSR31-1)	2.79	0.17	11.7	Kavir
	qLNS-7a	7	62	HvAMY2- GBMS0111	1.25 (GBMS0111)	4.05	−0.14	16.6	Badia
	qLNS-7b	7	98	Bmag0135- scssr07970	2.25 (Bmag0135)	4.47	0.16	18.1	Kavir
SCR	qSCS-1	1	126	ISSR16-2- CAAT1-A	0.41 (CAAT1-A)	3.87	−1.28	15.9	Badia
	qSCS-4	4	56	EBmac0906-EBmac0635	0.03 (EBmac0906)	2.85	−0.32	1.2	Badia
	qSCS-6a	6	62	ISSR31-1- Bmag0867	1.77 (ISSR31-1)	2.69	−0.51	11.4	Badia
	qSCS-6b	6	74	HVM65- EBmac0874	1.26 (EBmac0874)	2.70	−0.38	11.4	Badia
	qSCS-7	7	98	Bmag0135- scssr07970	2.25 (Bmag0135)	4.47	−0.48	18.1	Badia

## Data Availability

The data presented in this study are available upon request from the corresponding author.
